# Characterization and Drug Resistance Patterns of Ewing's Sarcoma Family Tumor Cell Lines

**DOI:** 10.1371/journal.pone.0080060

**Published:** 2013-12-02

**Authors:** William A. May, Rita S. Grigoryan, Nino Keshelava, Daniel J. Cabral, Laura L. Christensen, Jasmine Jenabi, Lingyun Ji, Timothy J. Triche, Elizabeth R. Lawlor, C. Patrick Reynolds

**Affiliations:** 1 Childrens Center for Cancer and Blood Diseases, Childrens Hospital Los Angeles, Los Angeles, California, United States of America; 2 Saban Research Institute, Childrens Hospital Los Angeles, Los Angeles, California, United States of America; 3 Department of Pediatrics, Keck School of Medicine, University of Southern California, Los Angeles, California, United States of America; 4 Department of Pathology, Keck School of Medicine, University of Southern California, Los Angeles, California, United States of America; 5 Departments of Pediatrics & Communicable Diseases and Pathology, University of Michigan School of Medicine, Ann Arbor, Michigan, United States of America; 6 Cancer Center and Departments of Cell Biology & Biochemistry, Pediatrics, and Internal Medicine, Texas Tech University Health Sciences Center School of Medicine, Lubbock, Texas, United States of America; University of Navarra, Spain

## Abstract

Despite intensive treatment with chemotherapy, radiotherapy and surgery, over 70% of patients with metastatic Ewing's Sarcoma Family of Tumors (EFT) will die of their disease. We hypothesize that properly characterized laboratory models reflecting the drug resistance of clinical tumors will facilitate the application of new therapeutic agents to EFT. To determine resistance patterns, we studied newly established EFT cell lines derived from different points in therapy: two established at diagnosis (CHLA-9, CHLA-32), two after chemotherapy and progressive disease (CHLA-10, CHLA-25), and two at relapse after myeloablative therapy and autologous bone marrow transplantation (post-ABMT) (CHLA-258, COG-E-352). The new lines were compared to widely studied EFT lines TC-71, TC-32, SK-N-MC, and A-673. These lines were extensively characterized with regard to identity (short tandem repeat (STR) analysis), p53, p16/14 status, and EWS/ETS breakpoint and target gene expression profile. The DIMSCAN cytotoxicity assay was used to assess *in vitro* drug sensitivity to standard chemotherapy agents. No association was found between drug resistance and the expression of EWS/ETS regulated genes in the EFT cell lines. No consistent association was observed between drug sensitivity and p53 functionality or between drug sensitivity and p16/14 functionality across the cell lines. Exposure to chemotherapy prior to cell line initiation correlated with drug resistance of EFT cell lines in 5/8 tested agents at clinically achievable concentrations (CAC) or the lower tested concentration (LTC): (cyclophosphamide (as 4-HC) and doxorubicin at CAC, etoposide, irinotecan (as SN-38) and melphalan at LTC; *P*<0.1 for one agent, and *P*<0.05 for four agents. This panel of well-characterized drug-sensitive and drug-resistant cell lines will facilitate *in vitro* preclinical testing of new agents for EFT.

## Introduction

Ewing's Family of Tumors (EFT) (Ewing's sarcoma (ES) and peripheral primitive neuroectodermal tumors (PNET)) are aggressive malignancies occurring in the childhood through adolescent/young adult years [1]. Ewing's sarcoma is the second most common primary bone cancer affecting children and young adults [2], [3] and is also among the most common soft tissue malignancies of this age group. Despite advances in the treatment of EFT that have led to survival rates of approximately 65–75% for localized disease, outcomes for patients with metastatic or recurrent EFT remain poor [1]–[3].

One dichotomy in EFT is between the dramatic chemoresponsiveness of primary tumors and the chemoresistance observed in most patients with metastases at diagnosis and in patients with localized disease which recurs. Though the mechanisms responsible for chemotherapy resistance in EFT have not been systematically studied, some disease-specific hypotheses may be entertained. A distinguishing feature of EFT is the universal presence of EWS/FLI1 (and related EWS/ETS) fusion transcription factors [4]. These oncogenic fusion transcription factors have been shown to alter the expression of a number of tumor promoting target genes, though none has yet been shown to correlate with clinical outcome [5], [6]. Despite this, one hypothesis for chemoresistance in EFT is that there is some difference in the expression pattern of these downstream loci which identifies or confers innate resistance, as has been postulated with osteosarcoma [7]. *TP53* mutations and alterations in p16/p14 function have been shown to influence therapeutic responsiveness in a variety of tumors and may be another cause of innate chemotherapy resistance. While most primary EFT have wild-type *TP53*, some have noted a higher incidence of treatment resistance in p53-non-functional EFT [8]–[10] or in EFT with alterations in p16/14 [11]–[13]. So the p53 or p16/14 status of EFT may also be hypothesized to explain some EFT treatment resistance. Finally, resistance to chemotherapy may be an acquired phenomenon, reflecting a selection of the tumor cell population best able to survive exposure to cytotoxic agents.

To address these potential mechanisms of resistance, we established a panel of cell lines from patients with EFT at various points during the course of their disease. Cell line genomic identity was validated and each was characterized with regard to their EWS/ETS fusion gene product, p53 and p16/14 status, and expression of EWS/ETS target genes of known or suspected biologic significance. We then characterized these lines with regard to their sensitivity to a number of common therapeutic agents employed in the treatment of clinical EFT.

## Methods

### EWS/ETS Breakpoint Characterization

This was determined by nested PCR amplification of cell line derived cDNA. Primers are listed in Table S1. The product of amplification was characterized by restriction endonuclease digestion as previously described [14]. As confirmation, selected lines also had their breakpoint PCR product subcloned into pcDNA 3.1 V5-his TOPO TA vector (Invitrogen) and sequenced.

### p53 and P16/14 status

Determination of p53 functionality was made by treating cell lines with etoposide (5 µg/ml) for 16 hours after which immunoblot and qPCR for p53 and p21 was conducted as previously described [15] (Table S2). Primers used are listed in Table S2 and antibodies were: p53 (Santa Cruz, clone D01), p21 Waf1/Cip1 (Cell Signaling Technology, clone DCS60). Status of p16/p14 was determined using quantitative genomic PCR as previously described [16].

### Gene Expression

Real time quantitative PCR was performed as previously described [15] using primers detailed in Table S1.

### Chemicals

Melphalan (L-PAM), carboplatin (CBDCA), topotecan (TPT), etoposide (ETOP), vincristine (VINC), and doxorubicin (DOX) were obtained from the Drug Synthesis & Chemistry Branch, Developmental Therapeutics Program, National Cancer Institute (Bethesda, MD). The active metabolite of irinotecan (SN-38) was from ABATRA Technology Co, Ltd, (Xi'an, 710075, China) and the active metabolite of cyclophosphamide (4-hydroperoxycyclophosphamide = 4-HC) was synthesized at Duke University, North Carolina and was a kind gift of Susan M. Ludeman and O. Michael Colvin. Fluorescein diacetate (FDA) was from the Eastman Kodak Company (Rochester, NY). Iscove's modified Dulbecco's medium (IMDM) was from BioWhittaker (Walkersville, MD). ITS™ Premix culture supplement (insulin, transferrin, and selenious acid) was from Collaborative Biomedical Products (Bedford, MA) and Eosin Y was from Sigma Chemical Co (St. Louis, MO).

### Cell Lines

The human EFT cell lines used in this study are listed in Table 1 along with their *in vivo* exposure to drugs in patients, the sites from which the specimens were obtained, the stage of the disease, the patient's age at diagnosis, and the doubling time (DT). For reference, A673 [17] and SK-N-MC [18] were originally classified as neuroblastoma cell lines in 1973 but have since been shown to be Ewing tumors [19], [20]. TC-32 [20], [21] and TC-71 [20] were originally described in the 1980's. CHLA-9, CHLA-10, CHLA-32, and CHLA-258 were originally described in the past decade [22]. CHLA-25 and COG-E-352 are newly described. All cell lines were maintained in Iscoves Modifed Dulbecco's Medium (IMDM), supplemented with L-glutamine (3 mM), insulin, and transferrin (5 µg/ml each), selenium (5 ng/ml), and 20% heat-inactivated FBS (whole medium) and were cultured at 37°C in a humidified incubator containing 95% room air plus 5% CO_2_ atmosphere. Cell lines were cultured without antibiotics so that *Mycoplasma* infection would not be masked and were tested and shown to be *Mycoplasma* negative. All cell lines used for this study except for A673 (which was not tested) were tested for viral pathogens by Research Animal Diagnostic Laboratory at the University of Missouri (Columbia, MO) and were negative for the following viruses: HIV1, HIV2, hepatitis A, hepatitis B, hepatitis C, Hantaan, Seoul, Sin Nombre, and lymphocytic choriomenengitis. Microscopic images of live EFT cell lines were captured using the Olympus IX71 Inverted Research Microscope, and visualized with QCapture Pro software from Qimaging [23].

**Table 1:** Characteristics and doubling time (DT) of 6 newly established and 4 previously characterized Ewing's Family of Tumor (EFT) cell lines.

Cell Line	Location	Sex	Age	Phase of Rx*	Chemotherapy prior to cell line established	DT (hours)
CHLA-9	Thoracic	F	14 y	DX	None	51
CHLA-10	Thoracic	F	14 y	Post-chemo	Cisplatin, Doxorubicin, Cyclophosphamide, Etoposide	32
CHLA-25	Unknown	F	2.6 y	Post-chemo	Etoposide, Ifosfamide, MENSA, Vincristine, Cyclophosphamide	99
CHLA-32	Pelvic	F	8.5 y	DX	None	26
CHLA-258	Lung Metastasis	F	14 y	Post-chemo	Post-Myeloablative Chemotherapy	89
COG-E-258	Peripheral blood. Post-mortem (fibula primary)	M	17 y	Post-chemo	Vincristine/Adriamycin/Cyclophosphamide. Alternating with Ifosfamide/Etoposide. Followed by High Dose Carboplatin/Ifosfamide/Etoposide. Followed by BMT.	28
TC-71	Humerus	M	22 y	Post-chemo	Derived from biopsy of locally recurrent tumor. Originally metastatic (1981)	21
TC-32	Ileum and adjacent soft tissue	F	17 y	DX	None (1979)	24
SK-N-MC	Retroorbital Metastasis	F	12 y	Post-chemo	Vincristine, Cyclophosphamide, Doxorubicin, Actinomycin (1968–1971)	23
A-673	Unknown	F	15 y		Unknown (1973)	25

* DX: cell lines established at diagnosis Post-chemo: after chemotherapy.

The cell lines SK-N-MC and A-673 were obtained from the American Type Culture Collection. All other cell lines were established in the laboratories of the authors (CPR or TJT) under protocols approved by the appropriate institutional Committee for Protection of Human Subjects (IRB). The COG-E-352 sample was obtained with written family consent from a post-mortem sample and was thus not established under an IRB-approved protocol as it was not human subject's research.

### Cytotoxicity assay

The cytotoxicity of 4-HC, L-PAM, CBDCA, TPT, ETOP, SN-38, VINC, and DOX was determined in 96-well plates using the semi-automated Digital Image Microscopy (DIMSCAN) system. DIMSCAN is a digital imaging system that measures the total fluorescence per well using digital thresholding with eosin Y quenching to eliminate background fluorescence and has a dynamic range of greater than 4 logs of cell kill [24], [25]. After overnight incubation (37°C, 5% CO_2_ with room air), various concentrations of chemotherapeutic drugs in 100 µl of complete medium were added to each well. The final drug concentrations were selected to include clinically achievable concentrations for each drug: 4-HC: 0–8 µg/ml, L-PAM: 0–10 µg/ml, CBDCA: 0–10 µg/ml, ETOP: 0–10 µg/ml, TPT: 0–100 ng/ml, SN-38: 0–24 ng/ml, VINC: 0–200 ng/ml, and DOX: 0–30 ng/ml [40]–[52]. Plates were assayed at 5 days after initiation of drug exposure by adding fluorescein diacetate (FDA) to the 96 well plates to a final concentration of 10 µg/ml, incubated for 30 min, followed by the addition of 50 µl of eosin Y (0.5% in normal saline). Fluorescence in each well proportional to the number of viable cells was then measured by DIMSCAN, and the results were expressed as the fractional survival of treated cells compared with untreated control cells [24], [25].

### Doubling Time

Doubling time (DT) (Table 1) was determined by counting triplicate 25 cm^3^ flasks with a hemocytometer (using trypan blue) every 2–3 days and then calculated with Microsoft Excel 2000 software, using the Y = X^o^*exp(B^c^) formula, where C is the time after seeding the cells, Y is the number cells after a period of time C, and X is the number of cells seeded at the time zero.

### Data Analysis

The mean number of logs of cell kill was calculated for each cell line at the lower tested concentration (LTC) and clinically achievable concentration (CAC) for each of the eight tested agents (Table 2). The association between drug sensitivity at the LTC and CAC and p53 functionality, p16/p14 status or phase of the cell lines was examined for each drug separately. Given that data from the same cell line were potentially correlated, those analyses were performed using the method of generalized linear equations with robust standard errors. Estimated difference in logs of cell kill and its 95% confidence interval (95% CI) are presented for each drug (Table 2). All P values reported are two-sided. Analyses were performed using STATA version 9.2. The concentration of drug that was cytotoxic and/or growth inhibitory for 99% of cells (IC_99_) (Table 3) was calculated using the software “Calcusyn” (Biosoft, Cambridge, UK).

**Table 2:** The log cell kill achieved at the lower tested concentration (LTC) and clinically achievable concentration (CAC).

Cell Line	4-HC µg/ml		L-PAM µg/ml		CBDCA µg/ml		ETOP µg/ml		TPT ng/ml		SN-38 ng/ml		DOX ng/ml		VINC ng/ml		Mean*	
	LTC 1.0	CAC 4.0	LTC 1.25	CAC 5.0	LTC 1.25	CAC 2.5	LTC 1.25	CAC 5.0	LTC 12.5	CAC 100	LTC 3	CAC 24	LTC 3.75	CAC 30	LTC 25	CAC 100	LTC	CAC
SK-N-MC	3.14	4.44	1.7	5.86	2.11	3.41	1.62	2.57	4.37	4.9	1.7	6.25	4.09	3.89	4.59	4.75	2.92	4.51
TC-71	3.85	5.39	0.83	5.15	−0.16	−0.21	1.37	1.83	5.59	6.35	0.83	4.7	2.5	6.42	3.66	3.57	2.31	4.15
TC-32	2.33	4.88	2.28	4.23	2.32	2.22	4.63	2.93	0.66	1.77	5.18	4.95	4.6	6.95	4.59	4.75	3.32	4.09
CHLA-9	1.53	2.67	1.47	2.59	0.1	0.27	3.54	3.63	3.03	4.36	3.36	3.81	4.12	6.08	1.74	1.72	2.36	3.14
COG-E-352	1.68	4.85	1.96	1.76	0.95	1.55	2.37	3.13	2.09	2.24	2.88	3.68	1.82	4.48	1.85	1.59	1.95	2.91
CHLA-32	1.56	3.68	1.63	2.4	1.15	1.78	2.45	2.42	2.65	3.27	3.03	3.6	2.18	3.75	1.4	1.33	2.01	2.78
CHLA-10	1.15	2.75	1.06	1.46	0.32	0.83	1.77	1.9	1.67	1.93	2.91	5.69	3.03	4.16	1.86	1.95	1.72	2.58
A-673	1.24	3.6	1.51	2.05	1.02	1.64	2.81	3.22	0.82	1.57	2.46	2.73	1.32	2.78	2.84	3.04	1.75	2.58
CHLA-258	0.77	1.12	0.35	1.01	0.52	0.61	0.75	0.76	1.1	1.5	1.13	1.55	2.73	3.4	2.13	2.21	1.19	1.52
CHLA-25	1.19	2.02	0.99	1.67	0.45	0.92	1.76	1.74	1.92	1.79	1.13	1.14	0.88	1.12	1.37	1.4	1.21	1.48
**Mean**	***1.8***	***3.54***	***1.3***	***2.8***	**.** ***88***	***1.3***	***2.3***	***2.4***	***2.3***	***2.9***	***2.4***	***3.8***	***2.7***	***4.3***	***2.6***	***2.6***	***2.08***	***2.97***

4-hydroperoxycyclophosphamide (4-HC), melphalan (L-PAM), carboplatin (CBDCA), doxorubicin (DOX), etoposide (ETOP), topotecan (TPT), irinotecan (as SN-38), and vincristine (VINC).

* Means were the mean numbers of logs of cell kill across all tested agents. Cell lines were sorted by the mean number of logs of cell kill at CAC.

**Table 3:** IC_99_ concentrations for each of the drugs tested with the EFT cell lines.

Cell Line	4-HC µg/ml	L-PAM µg/ml	CBDCA µg/ml	ETOP µg/ml	DOX ng/ml	TPT ng/ml	SN-38 ng/ml	DOX ng/ml	VINC ng/ml
SK-N-MC	1.5	2	2	4	6.5	7	.0.6	6.5	<0.1
TC-71	1	2	1	0.1	0.4	10	2	0.4	20
TC-32	1.3	0.6	>10	1	0.4	25	4.87	0.4	10
CHLA-9	1.6	1.8	>10	<0.01	0.7	3.1	0.01	0.7	>200
COG-E-352	1.5	>10	>10	1	7	0.01	1	7	>200
CHLA-32	1.6	2.5	5	>0.01	3	1.7	<0.001	3	66
CHLA-10	2.5	>10	>10	>10	3.5	>100	>24	3.5	>200
A-673	1.5	3.6	2.3	<0.1	11.8	40	<0.1	11.8	0.5
CHLA-258	>8	>10	>10	>10	0.9	>100	>24	0.9	0.2
CHLA-25	2.9	>10	>10	>10	>30	>100	>24	>30	>200

### DNA Fingerprinting

Short-tandem-repeat (STR) genotyping was performed to confirm the molecular identity of each cell line (Table S2) by polymerase chain reaction (PCR) using the AmpflSTR™ Identifiler™ kit according to the manufacturer's protocol (Applied Biosystems, Foster City, CA) [35] (www.COGcell.org/clid.php). The kit provides primers for the following microsatelite loci: D8S1179, D21S11, D7S820, CSF1PO, D3S1358, TH01, D13S317, D16S539, D2S1338, D19S433, vWA, TPOX, D18S51, D5S818, FGA, and includes the non-microsatellite, gender-specific amelogenin locus [26], [35]. Amplification was performed on the GeneAmp PCR System 9700 thermocycler (Applied Biosystems, Foster City, CA). PCR product was separated on a 3100 *Avant* Genetic Analyzer (Applied Biosystems, Foster City, CA) using POP-4 polymer and default conditions. Results were then analyzed using Genemapper ID version 3.2 software (Applied Biosystems, Foster City, CA). Results were visualized on electropherograms. CHLA-9 and CHLA-10 showed matching fingerprints and were considered validated since they were established from samples from the same patient acquired at separate times. CHLA-258 and COG-E-352 STR profiles matched corresponding patient material and the remaining cell line STR data showed unique signatures relative to all other cell lines in the COG cell line repository (http://strdb.cogcell.com) (Table S2).

### Ethics Statement

This research and consent process was reviewed and approved by the Human Subjects Protection Committees of Children's Hospital Los Angeles and Texas Tech University Health Sciences Center. Except for SK-N-MC and A-673 (obtained from the American Type Culture Collection) written informed consent was documented in the patient medical records. Cell lines TC-71 and TC-32 were established at the National Cancer Institute from biopsies taken to confirm the diagnosis. Both lines were established prior to HIPAA legislation and the consent utilized at the time allowed for unrestricted use of the lines for research and diagnosis. The cell lines CHLA-9, CHLA-10, CHLA-25, CHLA-32, CHLA-258 were established from samples obtained via written informed consent from parents and/or patients (as appropriate for age) and COG-E-352 was from a post-mortem blood sample obtained with written informed consent from the family of the patient in the laboratory of CP Reynolds, which is also the COG Cell Culture & Xenograft Repository (www.COGcell.org).

## Results

### Cell line clinical characteristics

The cell lines in the panel come from a wide diversity of clinical circumstances (Table 1). Some lines were derived from patients prior to receiving therapy or following lower intensity therapies used in earlier decades. Others were derived from heavily pretreated patients in an era of higher dose intensity. These include both patients treated with first-line chemotherapy and patients relapsing after myeloablative chemotherapy +/− radiotherapy supported by autologous stem cell transplant. The majority of clinical EFT are responsive to chemotherapy initially. This includes those presenting with metastatic disease, who have the poorest prognosis. Since one objective of establishing this panel is to enable evaluation of new therapies which may overcome drug resistance, the panel includes several lines from heavily pretreated patients. Four widely studied EFT lines are also included (TC-71, TC-32, SK-N-MC, A-673).

### Cell line molecular characteristics

A hallmark of Ewing's tumors is the presence of EWS/ETS chimeric transcription factors. This unique class of transcription factors is largely limited to Ewing's tumors, though some similar variants can be found uncommonly in leukemias [27]. Table 4 lists the EWS/ETS fusion product found in each of these lines. Since 85% of EFT primary tumors exhibit EWS/FLI1 fusions [4], it is not surprising that EWS/FLI1 is found in the majority of these cell lines. The 7/6 (or Type 1) EWS/FLI1 fusion is the most commonly found breakpoint in EFT primary tumors and is also the most common breakpoint in our cell line panel. Non-type 1 EWS/FLI1 fusions are also represented. While there had been some suggestion that these fusions may be associated with a poorer prognosis [28], [29], possibly due to distinct biologic features of these longer fusion products [30]–[32], this has not been found to be true in patients treated with more intensive therapies [33]. Two of the cell lines also harbor the EWS/ERG fusions, which are found in as many as 10% of primary tumors; such tumors have a presentation similar to EWS/FLI1 tumors [34] (Table 4). Overall, these cell lines accurately represent the spectrum of EWS/ETS rearrangements found in clinical cases.

**Table 4:** The EWS/FLI1 or EWS/ERG breakpoints and p53 and p16/14 status of EFT cell lines.

Cell Line	EWS	EXONS		Protein		
				p53	p16	p14
CHLA-9	FLI1	7	6	Functional	WT	WT
CHLA-10	FLI1	7	6	Non-Functional	WT	WT
CHLA-25	ERG	7	7	Non-Functional	WT	WT
CHLA-32	FLI1	7	6	Non-Functional	WT	WT
CHLA-258	FLI1	10	6	Functional	Null	Null
COG-E-352	ERG	7	8	Non-Functional	Null	Null
TC-71	FLI1	7	6	Non-Functional	Null	Null
TC-32	FLI1	7	6	Functional	Null	Null
SK-N-MC	FLI1	7	6	Non-Functional	WT	WT
A-673	FLI1	7	6	Non-Functional	Null	Null

As an additional characterization, we have performed STR analysis on the cell lines in our panel (Table S2). This will permit easy verification of cell line identity to future investigators. Such verification has been strongly advocated to ensure the integrity of results with cell lines and xenografts [35], [36].

### Independent prognostic covariates

**Figure 1:**
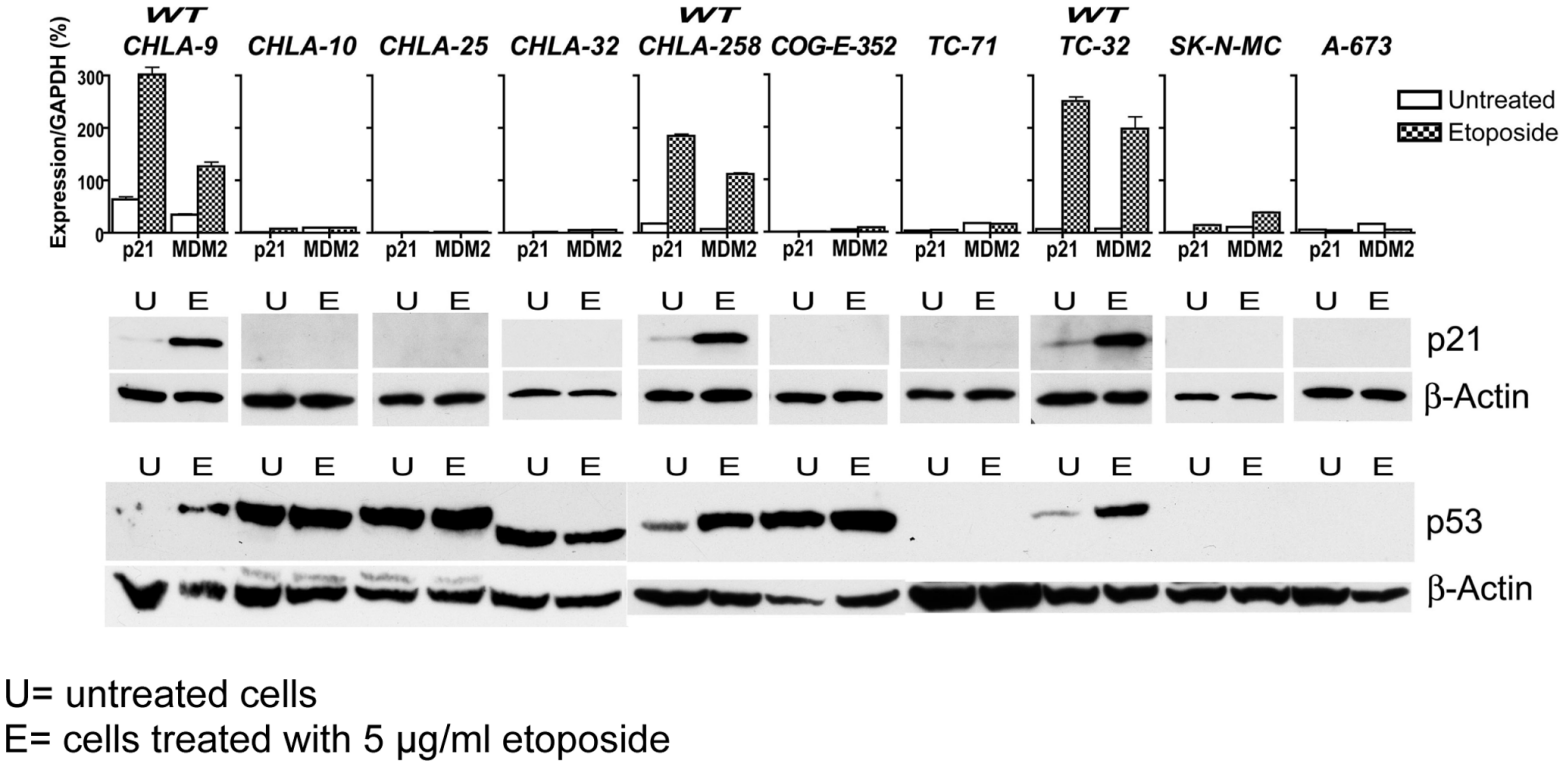
p53 status of EFT cell lines. Functional p53 status was measured by real time pPCR to assess induction of p53 target genes by exposure to etoposide, top panel. These results were corroborated by immunoblot for p21 and p53, lower panels.

In addition to fusion type, other covariates have been shown to significantly impact prognosis in EFT. While *TP53* mutations occur in only approximately 10% of EFT primary tumors [37], *TP53* mutations have been associated with a poor clinical outcome in several retrospective series [8]. Furthermore p16/14 status has also been associated with a poor outcome both independently and in combination with *TP53* mutations [12], [13]. Since these independent factors are of presumed significance, we assessed the status of each in the cell line panel. Functional p53 was assessed by induction of the p53 targets p21 and MDM2 mRNA in response to etoposide. We also documented p53 status by the induction of MDM2 and p53 protein by immunoblot. As shown in Figure 1, our cell line panel shows a high incidence of p53 loss of function (LOF) but three cell lines carried wild-type (functional) p53. Some lines with p53 LOF (CHLA-10, -25, -32 and COG-E-352) show abnormally high baseline levels of p53 protein by immunoblotting, as has been long observed with cells carrying a *TP53* mutation [10]. Other lines with documented deletions of *TP53* show an expected absence of p53 protein (e.g. SK-N-MC). Of note, the CHLA-9 cell line has functional p53, while the CHLA-10 line, established from a persistent focus of viable tumor after neo-adjuvant chemotherapy, is p53 non-functional. The p16/14 status of the cell lines (Table 4) was assessed by PCR of genomic DNA as has been described elsewhere [16]. We found a high proportion of these cell lines are p16/14 mutant when assessed for genomic deletion of the locus. These data are consistent with earlier studies of a partially overlapping panel of Ewing cell lines [38].

### Gene expression profile of cell lines

**Figure 2:**
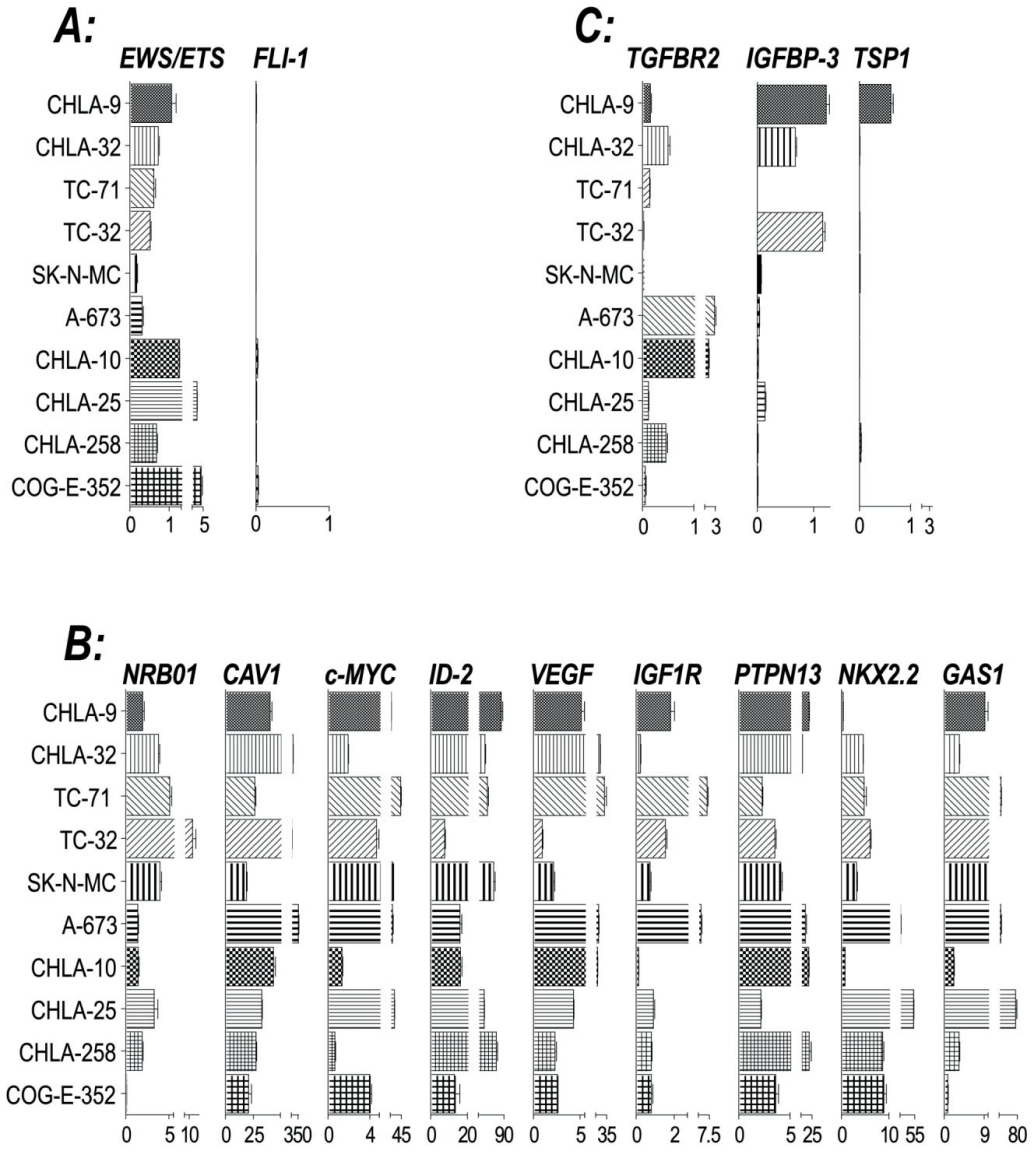
Gene expression survey of EFT cell lines. Expression as a percentage of the GAPDH transcript was assessed by real time quantitative PCR for the genes indicated. **Panel A** demonstrates the expression of EWS/ETS gene product along with native FLI-1. **Panel B** demonstrates high expression levels for a selection of loci known to be upregulated by EWS/ETS chimeric proteins. **Panel C** demonstrates low expression levels of loci shown to be downregulated by EWS/. ETS chimeric proteins.

The EWS/ETS family of fusion genes functions as aberrant transcription factors in EFT. The relative expression of these fusion gene products is shown in Figure 2A. This same panel also demonstrates limited expression of native FLI1 transcript as has been generally reported [39]. Many genes have been shown to be transcriptionally upregulated or downregulated by EWS/FLI1 and its homologues [5]. Several of these have been shown to be of biologic significance in EFT cell line models. Therefore, to further characterize our cell line panel, we evaluated several of these EWS/ETS target genes by real time quantitative PCR to see whether expression of our panel is typical of that reported in the literature. As can be seen in Figure 2B, a selection of upregulated EWS/ETS targets are expressed at generally high levels by our panel. Furthermore, a sample of transcriptionally downregulated genes are generally found to have low levels of expression (Figure 2C). Therefore, our panel is again generally typical of what would be expected from EFT cell lines. Those which stray from this pattern may be suitable for further mechanistic studies.

### Chemotherapy sensitivity profiles

**Figure 3:**
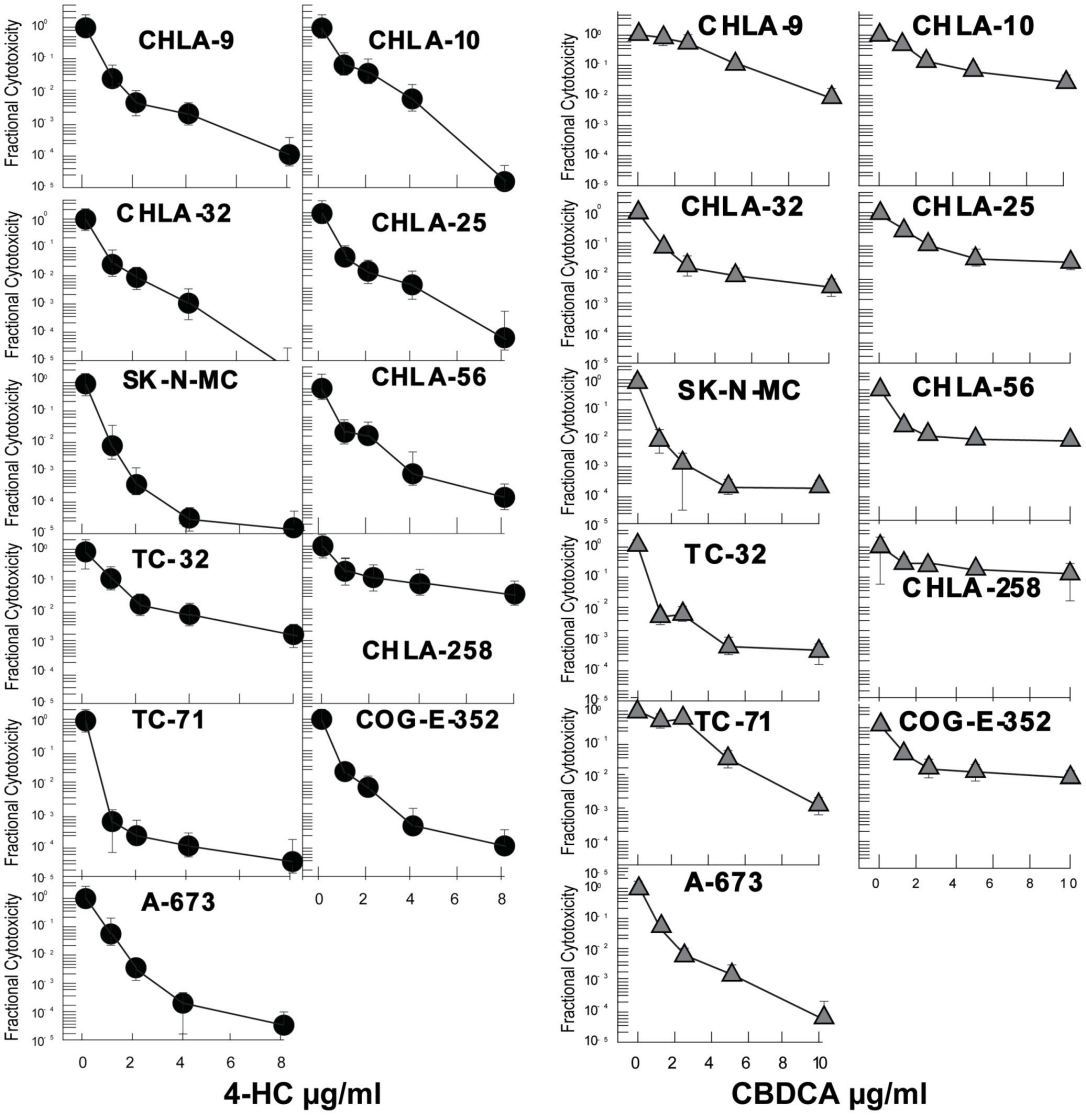
The EFT cell lines treated with chemotherapeutic agents. X-axis is drug concentration. The y-axis shows Fractional Cytotoxicity. 4-hydroperoxycyclophosphamide (4-HC: 0–8 µg/ml) and carboplatin (CBDCA: 0–10 µg/ml). Points represent the means ± SD.

The aim of this study was to establish a panel of *in vitro* EFT models for use in identifying drugs that are effective against chemotherapy-resistant EFT. Fractional cytotoxicity was measured in each cell line for our panel of antineoplastic agents using DIMSCAN. Plots for these agents are shown in Figure 3 and in Figure S1, S2, and S3. We defined a mid-range value from the clinically achievable range as the clinically achievable concentration (CAC) for each drug [40]–[52]. Resistance was defined for cytotoxic drugs commonly used in treating EFTs by comparing the average log cell kill by each drug at both the CAC and a lower tested concentration (LTC) (Table 2). Cell lines in Table 2 are listed in order of increasing chemotherapy resistance. On average, greater than two logs or more of cell kill are shown for all agents save carboplatin (CBDCA), which achieves average log cell kills of only 0.88 at LTC and 1.3 logs at CAC (Table 2). This is not surprising since the evidence for its clinical activity in EFT is not strong [53], though it has been used in combination regimens with some success [54].

The four lines demonstrating above average chemosensitivity (SK-N-MC, TC-71, TC-32, and CHLA-9 in Table 2) were either established in an era of lower chemotherapy intensity or were established at diagnosis, prior to exposure to chemotherapy. The remaining six lines include five of our newly characterized lines. They were established more recently, in an era of higher dose intensity, and include the two lines established after intensive myeloablative therapy and stem cell transplant (CHLA-258, COG-E-352). Furthermore, CHLA-10, established from a persistent tumor focus after neoadjuvant chemotherapy, is significantly more drug resistant than CHLA-9, which was established from the same patient at diagnosis. Thus, expression of a drug resistant phenotype in EFT cell lines appeared to increase with exposure to and intensity of chemotherapy given to the patients. A similar pattern is demonstrated by the IC_99_ concentrations for these same cell lines (Table 3).

Statistically, no consistent pattern was found in the association between drug sensitivity and p53 functionality or between drug sensitivity and p16/14 functionality across the cell lines. Two of the 8 tested agents (4-HC and doxorubicin) seemed to be more effective in p16/p14 null cell lines compared to wild type lines (Table 5). Doxorubicin showed significantly more cell kill in p53 functional lines at the LTC, but 4-HC displayed a higher relative cell kill in p53 non-functional lines.

**Table 5:** Differences and significance at lowest tested concentration (LTC) and clinically achievable concentration (CAC) in numbers of logs of cell kill between p53 non-functional vs. functional cell lines, between p16/14 null vs. wild lines, and between lines established at diagnosis vs. those established at PD or post-chemo.

	Lowest Tested Concentration (LTC)			Clinically Achievable Concentrations (CAC)		
Drugs	p-53 Status	p-16 Status	Phase Cell-Line Established	p-53 Status	p-16 Status	Phase Cell-Line Established
	Non-Functional *vs* Functional	Null *vs* Wild Type	At Diagnosis *vs* At PD/Post-chemo	Non-Functional *vs* Functional	Null *vs* Wild Type	At Diagnosis *vs* At PD/Post-chemo
4-HC	1.1(−0.34, 2.6) p = 0.13	0.87 (−0.54, 2.3) p = 0.23	0.55(−.73, 1.8) p = 0.40	2.6(0.91, 4.4) p = 0.003	2.0(0.94, 3.1) p<0.001	2.0(0.059, 3.9) p = 0.043
Etoposide	0.054(−1.5, 1.6) p = 0.94	0.36(−0.42, 1.1) p = 0.37	2.0(0.40, 3.6) p = 0.014	0.49(−1.1, 2.1) p = 0.55	0.052(−.92, 1.0) p = 0.92	1.3(−0.068, 2.6) p = 0.063
SN-38	0.12(−1.4, 1.6) p = 0.87	0.45(−.99, 1.9) p = 0.54	2.2(0.72, 3.7) p = 0.004	1.2(−0.76, 3.3) p = 0.22	0.14(−2.0, 2.3) p = .90	0.94(−1.8, 3.7) p = 0.51
Vincristine	.006(−1.6,1.6) p = 0.99	0.89(−.94, 2.7) p = 0.34	0.15(−1.9, 2.2) p = 0.89	−0.18(−1.9, 1.6) p = 0.84	0.75(−1.2, 2.7) p = 0.46	0.074(−2.1, 2.2) p = 0.95
Carboplatin	0.24(−0.77, 1.3) p = 0.64	0.26(−1.1, 1.6) p = 0.71	0.65(−0.39, 1.7) p = 0.22	0.54(−0.69, 1.8) p = 0.39	−0.16(−1.9, 1.6) p = 0.85	0.46(−0.81, 1.7) p = 0.48
Doxorubicin	−1.3(−2.3, −0.19) p = 0.022	−0.26(−1.5, 1.0) p = 0.70	0.45(−1.1, 2.0) p = 0.60	0.12(−2.3, 2.5) p = 0.92	1.9(0.074, 3.7) p = 0.041	2.0(−0.20, 4.3) p = 0.0.074
Melphalan	0.60(−.24, 1.4) p = 0.16	0.31(−0.40, 1.0) p = 0.39	1.0(0.19, 1.8) p = 0.016	1.2(−1.2, 3.5) p = 0.33	0.73(−1.8, 3.3) p = 0.57	0.96(−1.6, 3.5) p = 0.46
Topotecan	1.5(−0.63, 3.7) p = 0.16	0.11(−2.4, 2.6) p = 0.93	0.12(−1.3, 3.9) p = 0.88	1.3(−1.3, 3.9) p = 0.33	0.22(−2.7, 3.1) p = 0.88	0.68(−1.0, 2.4) p = 0.44

Confirming the above impression, a significant difference was observed between drug cytotoxicity and the phase of therapy when the EFT cell lines were established (at diagnosis versus after chemotherapy exposure) with a total of 5/8 tested agents at CAC or LTC (4-HC and doxorubicin at CAC, etoposide, SN-38 and melphalan at LTC; *P*<0.1 for one agent, and *P*<0.05 for four agents) (Table 5).

All drugs in this study except ETOP and VINC showed significantly more cell kill in average at the CAC than at the LTC. The high resistance to ETOP (CHLA-10, CHLA-25, COG-E-352), VINC (CHLA-25, COG-E-352) in the cell lines exposed to these drugs in patients suggest that etoposide and vincristine may have limited activity in recurrent EFT. There was no apparent correlation observed in the cytotoxicity of the drugs and expression of EWS/FLI1 regulated genes.

## Discussion

Over the past two decades, chemotherapy intensity of EFT treatment protocols has steadily increased. The standard chemotherapy for localized EFT includes vincristine, ifosfamide, doxorubicin and etoposide (VIDE) in Europe or vincristine, doxorubicin, cyclophosphamide, ifosfamide and etoposide (VDC-IE) in North America [1], [55]. High-dose (myeloablative) chemotherapy with autologous stem cell rescue has been used to further intensify therapy in EFT [56]–[59] in some poor-risk populations. However, those with metastatic disease at presentation have a much worse outcome, with an approximately 10–30% 5-year event-free survival rate [1], [60], regardless of which therapy is employed. In some cases, attempts to further intensify therapy of known effective agents have met with an alarmingly high incidence of secondary malignancy [61]. Developing new therapeutic strategies and identifying new effective chemotherapeutic agents is critical to improving the outcome for those with metastatic or recurrent EFT.

The findings reported in this paper provide an important resource in this regard. A subset of the cell lines are in use by the National Cancer Institute Pediatric Preclinical Testing Program [62], [63] (www.PPTPinvitro.org; http://pptp.nchresearch.org/). The cell line panel characterized in this paper can be readily obtained from the Children's Oncology Group (COG) Cell Line and Xenograft Repository (www.COGcell.org). Such a public repository and its associated STR database facilitates biological and preclinical therapeutic studies by helping to avoid discrepancies that may result from differences in cell lines acquired as a result of prolonged passage or contamination. The comprehensive data presented in this paper on EWS/ETS breakpoint, on p53 and p16/14 status, and on expression of EWS/ETS target loci improves the utility of these readily available cell lines for EFT translational studies.

Importantly, we have also characterized this cell line panel with regard to baseline chemosensitivity. These data reveal several important aspects of common EFT cell lines. First, many of the commonly employed EFT cell lines are relatively drug sensitive. While most EFT are drug sensitive at diagnosis, resistant disease emerges in 30% of patients with localized disease and in 70–80% of patients with metastases. As chemotherapy-resistant EFT patients are rarely salvaged with any known therapy, the key to improving outcome is to understand the mechanisms present in resistant cells that lead to treatment failure and to employ models of such resistant cells in laboratory studies seeking novel therapies. To simply study the same long-established drug-sensitive lines does not adequately address the problem of resistant disease. Therefore, the newer, multi-drug resistant lines in this panel will provide an important new resource for preclinical therapeutic studies in EFT.

We have also observed that there appears to be no obvious correlation between the EWS/FLI1 or EWS/ETS fusion genes, their gene target expression, and drug resistance. Certainly, one cannot conclude from this limited study that an EWS/FLI1 target gene set predicting resistance does not exist. However, the identification of such a predictive gene expression pattern will depend on more extensive gene expression profiling studies and will likely require an even larger panel of well-characterized cell lines. Furthermore, since the majority of EFT at presentation are drug-sensitive, profiling strategies of primary tumors may fail to identify the roots of chemoresistance that are present in a small subpopulation of the tumor.

Our data did not demonstrate a consistent pattern in the association between drug sensitivity and p53 or p16/14 status, which is not consistent with the previously cited observations based on clinical outcomes [8]–[13]. The small sample size in our study may be insufficient in this regard. Furthermore, since 90% of presenting EFTs are *TP53* wild-type, loss of function for p53 does not explain the majority of treatment failures, unless p53 loss-of-function emerges during treatment as was the case for the CHLA-9/10 pair.

Finally, we observe that a history of chemotherapy exposure prior to cell line establishment correlated with drug resistance. This is consistent with clinical observations and has also been observed in neuroblastoma cell lines [41], suggesting that treatment resistance emerges as an adaptation to survival in the face of cytotoxic therapy. Whether the resistance is merely selected for or is acquired in the course of therapy due to mutations and/or epigenetic alterations in the tumor cell population (induced or selected for) is unknown.

The cell lines described here provide a well characterized panel of cell lines for preclinical studies aimed at identifying new active agents in EFT that can be obtained at www.COGcell.org. They should prove helpful in conducting preclinical studies to support the design of novel clinical strategies to overcome drug resistance developed during the course of treatment by identifying agents active against drug-resistant tumor cells.

## Supporting Information

Figure S1 **Fractional cytotoxicity of EFT cell lines treated with irinotecan as SN-38 (0–24 ng/ml) and vincristine (VINC: 0–200 ng/ml).**
(TIF)

Figure S2 **Fractional cytotoxicity of EFT cell lines treated with melphalan (L-PAM: 0–10 µg/ml) and etoposide ETOP (0–10 µg/ml).**
(TIF)

Figure S3 **Fractional cytotoxicity of EFT cell lines treated with topotecan (TPT: 0–100 ng/ml) and doxorubicin (DOX: 0–30 ng/ml).**
(TIF)

Table S1 PCR Primers. A. Primers used for gene expression. B. Primers used for p53 testing.
(DOC)

Table S2 Short tandem repeat (STR) profiles of EFT cell lines.
(DOC)
