# Supplementary figures and images for: Characterization and Drug Resistance Patterns of Ewing's Sarcoma Family Tumor Cell Lines
Source: PLoS One. 2013 Dec 2;8(12):e80060. doi: 10.1371/journal.pone.0080060 (PMC3846563; doi:10.1371/journal.pone.0080060)

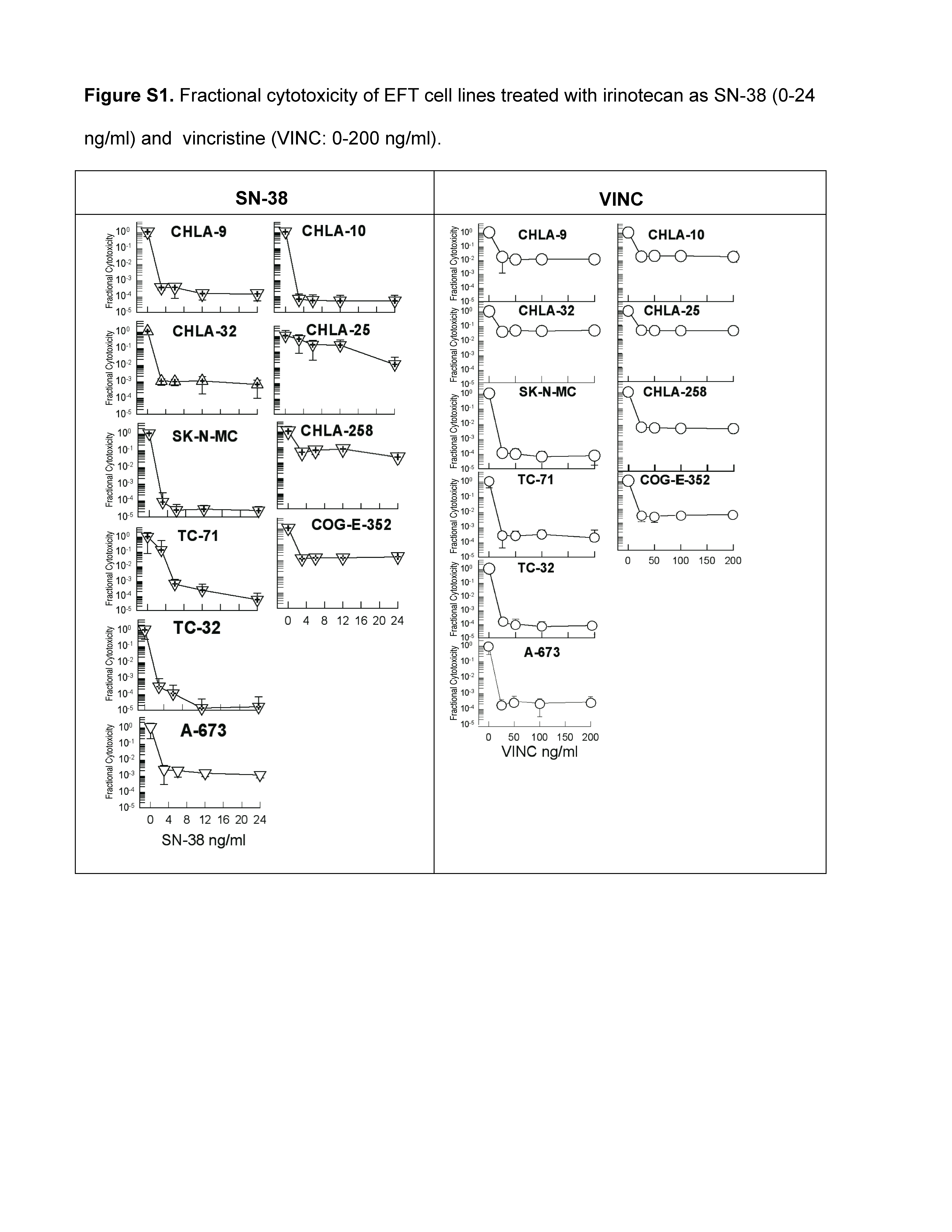

Supplement: Figure S1 — Fractional cytotoxicity of EFT cell lines treated with irinotecan as SN-38 (0–24 ng/ml) and vincristine (VINC: 0–200 ng/ml). (TIF) [file pone.0080060.s001.tif]

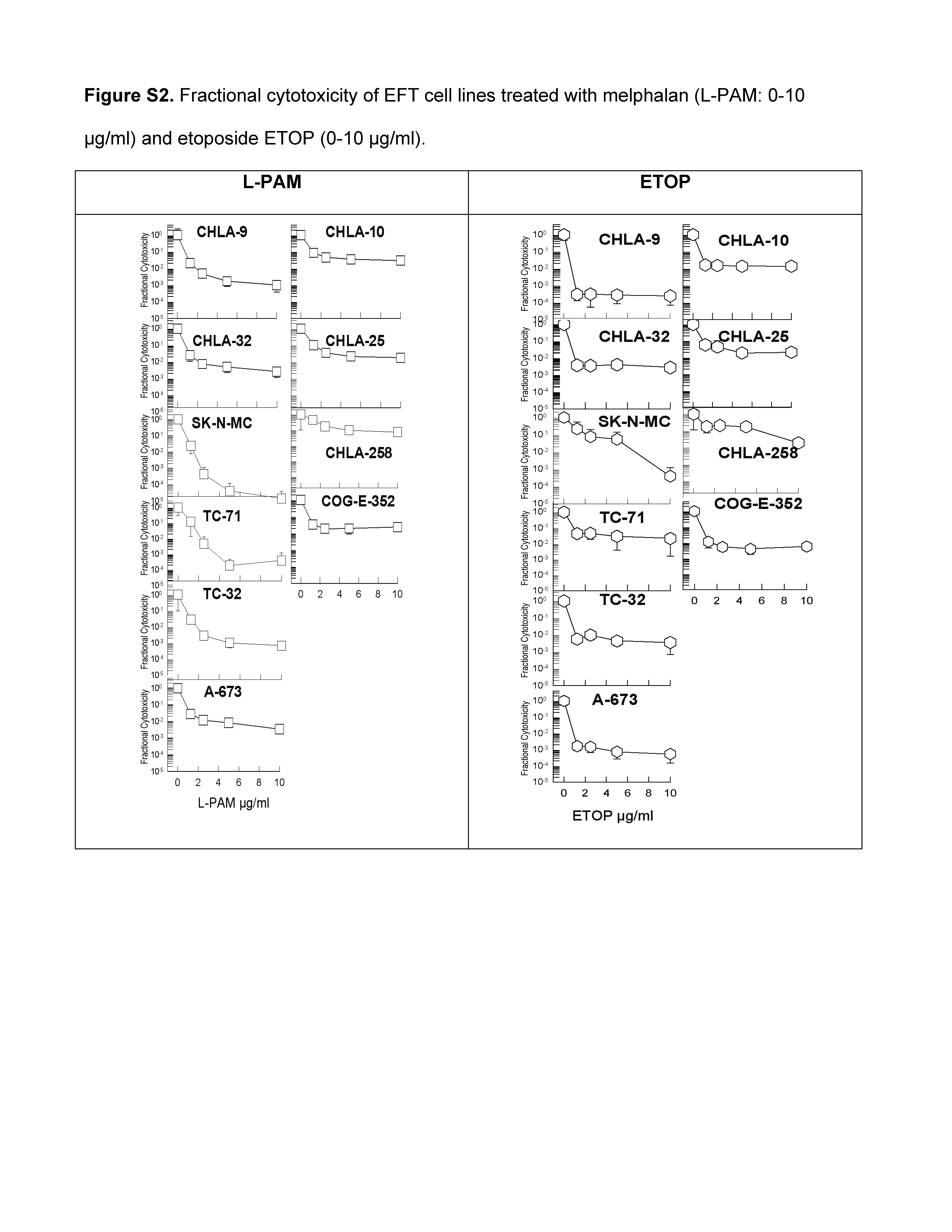

Supplement: Figure S2 — Fractional cytotoxicity of EFT cell lines treated with melphalan (L-PAM: 0–10 µg/ml) and etoposide ETOP (0–10 µg/ml). (TIF) [file pone.0080060.s002.tif]

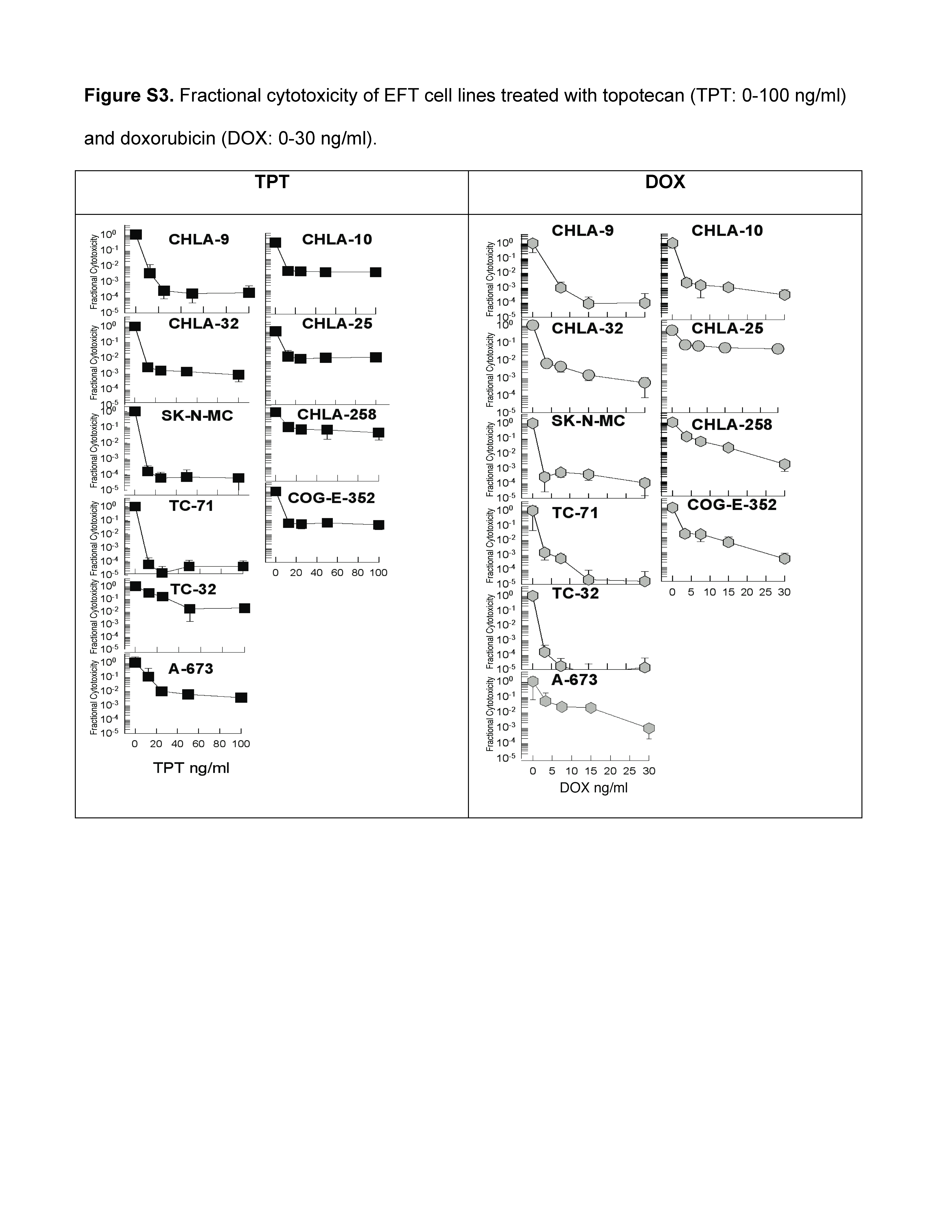

Supplement: Figure S3 — Fractional cytotoxicity of EFT cell lines treated with topotecan (TPT: 0–100 ng/ml) and doxorubicin (DOX: 0–30 ng/ml). (TIF) [file pone.0080060.s003.tif]
