# Supplementary material for: Characterization and Drug Resistance Patterns of Ewing's Sarcoma Family Tumor Cell Lines
Source: PLoS One. 2013 Dec 2;8(12):e80060. doi: 10.1371/journal.pone.0080060 (PMC3846563; doi:10.1371/journal.pone.0080060)
Supplement: Table S1 — PCR Primers. A. Primers used for gene expression. B. Primers used for p53 testing. (DOC) [file pone.0080060.s004.doc]

**Table S1. PCR Primers**

**A. Primers used for gene expression**

| **Gene** | **Forward Primer** | **Reverse Primer** |
| --- | --- | --- |
| FLI1 | ggc cat tct tct cgt cca ta | atc agc cag tga ggg tca ac |
| c-MYC | TTC GGG TAG TGG AAA ACC AG | CAG CAG CTC GAA TTT CTT CC |
| CAV1 | GAG CTG AGC GAG AAG CAA GT | CAA ATG CCG TCA AAA CTG TG |
| VEGF-a | AAG GAG GAG GGC AGA ATC AT | ATC TGC ATG GTG ATG TTG GA |
| ID-2 | ATC CCC CAG AAC AAG AAG GT | AAT TCA GAA GCC TGC AAG GA |
| NRB01 | AGG GGA CCG TGC TCT TTA AC | ATG ATG GGC CTG AAG AAC AG |
| IGF1R | GTC CAG GCC AAA ACA GGA TA | CAG AGG CAT ACA GCA CTC CA |
| PTPN13 | GAC CTG GAG TTC AGC TTT GC | GAG CCA TAT CCG GTG GTA GA |
| NKX2.2 | GAA CCC CTT CTA CGA CAG CA | GGG TCT CCT TGT CAT TGT CC |
| GAS1 | GAA GGG ATG GTT GGG GAT AC | GCA GAC GAG TTG GGA GTT TC |
| TGFBR2 | TTT TCC ACC TGT GAC AAC CA | GGA GAA GCA GCA TCT TCC AG |
| IGFBP-3 | CCT GCC GTA GAG AAA TGG AA | GGC TGC CCA TAC TTA TCC AC |
| TSP1 | TTG TCT TTG GAA CCA CAC CA | CTG GAC AGC TCA TCA CAG GA |
| EWS-FLI1 7-6 (aka ESBP, and for 7-5 breakpoints, including CHLA- 9, 10, 32, 218, TC-71, TC-32, A-673, SK-N-MC) | CGA CTA GTT ATG ATC AGA GCA GT | CCG TTG CTC TGT ATT CTT ACT GA |
| EWS-FLI1 10-6 (used for CHLA-258) | GAA GAG GGG GAT TTG ATC GT | CCA AGG GGA GGA CTT TTG TT |
| EWS-ERG (used for CHLA-25, COG-E-352) | ATC CTA CAG CCA AGC TCC AA | GCA CTG TGG AAG GAG ATG GT |
| GAPDH | TCC TCT GAC TTC AAC AGC GAC A | ATG GTA CAT GAC AAG GTG CGG |

| **Gene** | **Forward Primer** | **Reverse Primer** |
| --- | --- | --- |
| p53 | GTT CCG AGA GCT GAA TGA GG | TCT GAG TCA GGC CCT TCT GT |
| p21 | GGA AGA CCA TGT GGA CCT GT | GGA TTA GGG CTT CCT CTT GG |
| MDM2 | GTA TCA GGC AGG GGA GAG TG | GAA GCC AAT TCT CAC GAA GG |
| GAPDH | TCC TCT GAC TTC AAC AGC GAC A | ATG GTA CAT GAC AAG GTG CGG |

**B. Primers used for p53 testing**
