# Supplementary material for: Characterization and Drug Resistance Patterns of Ewing's Sarcoma Family Tumor Cell Lines
Source: PLoS One. 2013 Dec 2;8(12):e80060. doi: 10.1371/journal.pone.0080060 (PMC3846563; doi:10.1371/journal.pone.0080060)
Supplement: Table S2 — Short tandem repeat (STR) profiles of EFT cell lines. (DOC) [file pone.0080060.s005.doc]

**Table S2. Short tandem repeat (STR) profiles of EFT cell lines.**

| **STR Locus** | **CHLA-9** | **CHLA-10** | **CHLA-25** | **CHLA-32** | **CHLA-258** | **COG-E-352** | SK-N-MC | **TC-32** | TC-71 | A-673 |
| --- | --- | --- | --- | --- | --- | --- | --- | --- | --- | --- |
| D8S1179 | 10,13 | 10,13 | 13,15 | 13,15 | 14,15 | 13,14 | 10 | 12 | 10,12 | 11,13 |
| **D21S11** | 28,29 | 28,29 | 29,32.2 | 28,31 | 31.2 | 29,30 | 30,31.2 | 27,28 | 29,30 | 29,30.2 |
| **D7S820** | 9,10 | 9,10 | 8,10 | 8,9 | 11,12 | 8,9 | 8 | 8,11 | 10 | 10,12 |
| **CSF1PO** | 12 | 12 | 10 | 10,11 | 10,11 | 10,11 | 10 | 11,13 | 10,11 | 11,12 |
| **D3S1358** | 15,16 | 15 | 17,18 | 16 | 15 | 17,18 | 15,16 | 15,16 | 15,17 | 14 |
| **TH01** | 7,9.3 | 7 | 7 | 6,9.3 | 7,9.3 | 6,7 | 9.3 | 6,9.3 | 9.3 | 9.3 |
| **D13S317** | 11,12 | 12 | 10,12 | 12 | 9,11 | 8,11 | 11 | 10,12 | 11,12 | 8,13 |
| **D16S539** | 11 | 11 | 11,12 | 12 | 10,12 | 9 | 12 | 13,14 | 11,14 | 11 |
| **D2S1338** | 19,20 | 20 | 19,22 | 24 | 17,23 | 19,24 | 17,21 | 17,19 | 20,24 | 16,21 |
| **D19S433** | 12,13 | 12,13 | 14 | 13,15 | 14 | 14,14.2 | 13,15.2 | 12,14 | 14,16.2 | 13,14 |
| **vWA** | 16,17 | 16 | 15,17 | 16,17 | 16,18 | 18,20 | 17,18 | 15,18 | 17 | 15,18 |
| **TPOX** | 8,11 | 11 | 8 | 8,10 | 8,11 | 8,11 | 9,11 | 9,11 | 8,9 | 8 |
| **D18S51** | 12,14 | 12,14 | 14,17 | 18,19 | 12,17 | 12,14 | 13,14 | 11,19 | 12,15 | 13,16 |
| **AMEL** | X | X | X | X | X | X,Y | X | X | X,Y | X |
| **D5S818** | 12 | 12 | 10 | 11 | 7,11 | 10,13 | 11 | 12,13 | 10 | 11,12 |
| **FGA** | 23,24 | 23 | 24,25 | 24 | 19,25 | 19,21 | 21,25 | 23,24 | 24,26 | 19,20 |
